# Factors associated with cervical cancer screening participation in Southwest China: a retrospective database-based cross-sectional study

**DOI:** 10.3389/fmed.2026.1740068

**Published:** 2026-02-11

**Authors:** Junjun Xiong, Yan Qian, Maoshi Wang, Xingyu Sun, Jin He

**Affiliations:** 1 Department of Health Management Center, Luzhou Maternal and Child Health Hospital, Luzhou Second People's Hospital, Luzhou Women and Children's Hospital, Luzhou, Sichuan, China; 2 Department of Gynecology, Luzhou Maternal and Child Health Hospital, Luzhou Second People's Hospital, Luzhou Women and Children's Hospital, Luzhou, Sichuan, China; 3 Department of Pathology, Luzhou Maternal and Child Health Hospital, Luzhou Second People's Hospital, Luzhou Women and Children's Hospital, Luzhou, Sichuan, China; 4 Department of Gynecology, The Affiliated Traditional Chinese Medicine Hospital, Southwest Medical University, Luzhou, Sichuan, China

**Keywords:** cervical cancer, healthcare accessibility, retrospective database study, screening participation, socioeconomic disparities, Southwest China

## Abstract

**Background:**

Cervical cancer remains a major public health concern in developing regions, including Southwest China, where population-based screening coverage remains limited. This study aimed to identify sociodemographic, behavioral, and healthcare-related factors associated with cervical cancer screening participation among women in this region through a retrospective, database-based cross-sectional analysis.

**Methods:**

De-identified data from 2,320 women aged 20–65 years were retrospectively retrieved from institutional health information systems and standardized regional screening databases covering January–December 2023. Screening participation was defined as documentation of a Pap smear or HPV test within the preceding 3 years. Variables on demographic characteristics, health behaviors, and healthcare accessibility were analyzed using chi-squared tests, t-tests, and multivariable logistic regression. Subgroup analyses stratified by residence (urban vs. rural) and sensitivity analyses under alternative model assumptions were conducted to assess consistency and robustness. No new data were collected, and no direct participant contact occurred.

**Results:**

Younger age (adjusted odds ratio [aOR] = 0.96 per year; 95% confidence interval [CI]: 0.94–0.98), higher education (aOR = 2.30; 95% CI: 1.84–2.87), and higher income (aOR = 1.74; 95% CI: 1.44–2.11) were independently associated with greater screening participation. Urban residence (aOR = 2.13; 95% CI: 1.78–2.54), medical insurance coverage (aOR = 4.12; 95% CI: 3.19–5.33), and shorter distance to healthcare facilities (aOR = 0.39 for >15 km; 95% CI: 0.28–0.54) were also strongly associated with screening participation. Non-smoking and regular physical activity were positively associated with participation. Patterns were consistent across urban and rural subgroups, with no significant interaction effects.

**Conclusion:**

This retrospective database analysis identified socioeconomic and healthcare accessibility disparities that were associated with cervical cancer screening participation in Southwest China. Targeted interventions that strengthen education, expand insurance coverage, and improve access to primary screening services are critical to increasing screening uptake and advancing cervical cancer prevention equity.

## Introduction

Cervical cancer remains a major global public health concern, particularly in low- and middle-income countries (LMICs), where access to preventive screening and timely treatment is limited. It is the fourth most common cancer among women worldwide, with approximately 604,000 new cases and 342,000 deaths reported in 2020, the majority occurring in resource-limited settings (1). Persistent infection with high-risk human papillomavirus (HPV) is the primary etiological factor for cervical cancer, making HPV vaccination and regular screening critical strategies for early detection and prevention (2–4). However, substantial disparities persist in cervical cancer screening uptake, particularly in rural and socioeconomically disadvantaged communities. Understanding multilevel factors associated with screening participation is important for informing discussions around strategies to improve screening coverage and cervical cancer prevention.

China, despite achieving improvements in healthcare access, continues to exhibit disparities in cervical cancer screening, particularly in less developed regions such as Southwest China. The Chinese government has implemented national cervical cancer screening programs, including the “Two Cancers Screening Program” for rural women, which provides free or subsidized Pap smear and HPV testing services (5, 6). In rural areas, the government-funded “Two Cancers Screening Program” provides cervical cancer screening services free of charge for eligible women. In contrast, women in urban settings who undergo self-initiated screening at hospital outpatient clinics typically pay a modest out-of-pocket fee (approximately 50–120 CNY, depending on facility and test type), which is partially reimbursed through the basic medical insurance system. These differences in screening cost and reimbursement may contribute to the observed socioeconomic disparities in screening participation. However, screening participation remains suboptimal, with significant geographic and socioeconomic variations (7, 8). Previous studies have identified several barriers to screening, including financial constraints, inadequate awareness, cultural beliefs, and limited access to healthcare facilities, particularly in rural areas (9, 10). In addition, factors such as age, education, employment status, and health insurance coverage have been shown to influence screening behaviors (11, 12). Nevertheless, a comprehensive analysis integrating sociodemographic, behavioral, and healthcare accessibility factors within this specific regional context remains limited.

Southwest China is characterized by significant ethnic diversity, economic disparities, and varied healthcare infrastructure, all of which may contribute to inequities in cervical cancer screening. Rural residents often experience greater challenges in accessing preventive healthcare services due to geographic remoteness and limited availability of medical resources (13). Additionally, cultural and linguistic differences among ethnic minorities may further exacerbate disparities in screening uptake (14). While urban populations generally have better access to healthcare facilities, other socioeconomic barriers, such as financial limitations and competing health priorities, may still hinder participation in screening programs. Therefore, a nuanced understanding of these factors is necessary to inform targeted interventions that can enhance screening coverage in both urban and rural settings. In Southwest China, cervical cancer screening is primarily delivered through community-based primary healthcare centers and township hospitals under the supervision of the local Maternal and Child Health (MCH) institutions. In urban areas, most women are screened at hospital-affiliated outpatient clinics, whereas in rural counties, screening is provided by trained nurses and general practitioners at township-level clinics as part of the government-supported “Two Cancers Screening Program.”

To address these knowledge gaps, this study aims to examine multilevel factors associated with cervical cancer screening participation among women in Southwest China. By analyzing sociodemographic characteristics, health behaviors, and healthcare accessibility, this study seeks to examine factors associated with higher or lower screening participation. The findings will provide valuable insights for policymakers and healthcare professionals to develop tailored strategies that promote equitable access to cervical cancer screening and ultimately contribute to reducing the burden of cervical cancer in this region.

## Methods

### Study design

This study was a retrospective, cross-sectional investigation that reviewed and analyzed existing medical and screening records of women who had undergone cervical cancer screening between January and December 2023 in Southwest China. All information was obtained from pre-existing electronic health information systems and standardized screening databases maintained by local health authorities. No new data were prospectively collected, and no follow-up, clinical intervention, or participant contact was performed. The study aimed to identify sociodemographic, behavioral, and healthcare-related factors associated with cervical cancer screening participation among adult women.

### Setting

The analysis was conducted in Southwest China, a region characterized by diverse urban–rural populations and pronounced socioeconomic heterogeneity. Cervical cancer screening in this area is routinely carried out through a regional public health network, which provides Pap smear and HPV testing under national screening guidelines. Data for this retrospective study were extracted solely from administrative and clinical databases covering January 1–December 31, 2023. These databases systematically record demographic characteristics, screening participation, and health indicators documented during routine outpatient or community health visits. Accordingly, the analytic sample represents women with documented encounters captured by routine health service databases and does not fully include women who had no contact with the healthcare system during the study period. No active data collection or recontact of participants was performed, ensuring the purely retrospective nature of the study.

### Participants

Eligible participants were women aged 20–65 years with documented health records and screening-related information in the regional health information system during 2023. Inclusion required continuous residence in the study region for ≥1 year and complete demographic and screening data. Exclusion criteria were: prior cervical cancer diagnosis, hysterectomy, severe or acute illness, pregnancy, cognitive or psychiatric disorder, or missing data on key variables. After applying these criteria, 2,320 valid anonymized records were included. Because all data were derived retrospectively from existing databases, no recruitment, follow-up, or patient interviews were conducted.

### Variables

In this retrospective database analysis, the primary outcome variable was cervical cancer screening participation, defined as documentation of a Pap smear or high-risk HPV test within the preceding 3 years, consistent with national screening guidelines. All explanatory variables were extracted directly from pre-existing fields in the electronic medical and screening databases without remeasurement or modification. Sociodemographic characteristics included age, ethnicity (Han or ethnic minority), education level (primary or below, secondary, or college and above), monthly household income (<3,000 CNY, 3,000–6,000 CNY, or >6,000 CNY), and place of residence (urban or rural). Behavioral information, derived from routine health examination records, comprised smoking status (non-smoker, former, or current) and physical activity level (none, 1–2 times per week, or ≥3 times per week). Healthcare accessibility indicators included medical insurance coverage (insured or uninsured) and the linear distance from the participant’s home to the nearest healthcare facility (≤5 km, 6–15 km, or >15 km), as documented in administrative registration files. In addition, a clinical variable representing a documented history of sexually transmitted infections (STIs) was obtained from diagnostic entries in the electronic health record. Potential confounders such as marital status and parity, also available in the database, were included in sensitivity analyses to assess the robustness of the multivariable models.

### Data sources and measurement

All information originated from validated electronic health information systems and standardized cervical cancer screening databases maintained by local health authorities. Data had been collected previously during routine preventive or outpatient visits for administrative and clinical purposes. Screening status was verified through documented results of Pap or HPV tests. Quality control procedures—including duplicate record removal, cross-checking, and completeness validation—were conducted by trained data managers under epidemiologist supervision. No new data collection, participant interviews, or follow-up assessments occurred during this analysis.

### Bias control

To minimize potential bias inherent to retrospective research, several methodological safeguards were implemented. Selection bias was reduced by including all eligible women who met the predefined inclusion criteria and had complete medical and screening records within the study period, thereby ensuring comprehensive data coverage. Information bias was minimized through standardized data extraction and uniform coding procedures applied to validated electronic health databases, which maintained consistency across all variables. To further limit observer bias, analysts responsible for data cleaning and statistical modeling were blinded to the specific study hypotheses. Moreover, because all information originated from historical, electronically documented records rather than self-reported data, recall and interviewer bias were inherently avoided.

### Study size

This was a census-based retrospective analysis, including all eligible records from 2023 rather than a sampled cohort; thus, no *a priori* sample size calculation was required. A *post hoc* power analysis indicated that the available sample (*n* = 2,320) provided >90% power to detect moderate effect sizes (*α* = 0.05, two-sided) in multivariable logistic regression with up to 10 predictors, confirming adequate statistical precision.

### Quantitative variables

Age and distance to the nearest healthcare facility were treated as continuous variables. Income and physical activity level were analyzed as ordered categorical variables. All continuous variables were checked for normality, and non-normal distributions were log-transformed when necessary. No new measurements or re-collection of variables were performed; all values originated from the retrospective databases.

### Statistical methods

All analyses were performed using IBM SPSS Statistics version 28.0. Continuous variables were summarized as mean ± SD and compared by t-tests; categorical variables as frequencies (%) and compared using *χ*^2^ tests. To identify independent predictors of screening participation, multivariable binary logistic regression was applied. Results were reported as adjusted odds ratios (aORs) with 95% confidence intervals. Confounders were included simultaneously in the regression model, and multicollinearity was excluded (VIF < 2.5). Model calibration was evaluated with the Hosmer–Lemeshow test. Subgroup analyses (urban vs. rural) and interaction tests assessed potential effect modification by residence. Missing data (<5%) were handled via multiple imputation with chained equations (20 imputations). Sensitivity analyses included (1) complete-case analysis, (2) inclusion of additional covariates (marital status, parity), and (3) model coefficient comparisons across imputed datasets. All tests were two-tailed, with statistical significance defined as *p* < 0.05. Given the retrospective design, the analysis focused on associations rather than causal inference.

## Results

### Baseline characteristics of the study population (*N* = 2,320)

Table 1 summarizes the baseline characteristics of women included in the analysis, stratified by cervical cancer screening participation status. The mean age of all participants was 38.5 years (SD 8.6). Screened women were statistically younger than those who were not screened (37.8 vs. 39.8 years, *p* < 0.001), although the absolute difference of 2.0 years was small and likely of limited clinical relevance. A greater proportion of screened women were aged <30 years (20.7% vs. 13.8%) or 30–39 years (42.1% vs. 35.6%), whereas women aged ≥50 years were less frequently screened (9.0% vs. 14.9%, *p* < 0.001). In terms of sociodemographic characteristics, Han ethnicity was associated with a higher likelihood of screening compared with ethnic minority status (84.1% vs. 73.6%, p < 0.001). Both education level and monthly income, as recorded in the health information system, showed strong positive associations with screening participation. Women with college or higher education had the highest screening proportion (31.0%), followed by those with secondary (46.9%) and primary education or below (22.1%, *p* < 0.001). Screening participation was most frequent among women with a monthly income of 3,000–6,000 CNY (51.7%), followed by those earning <3,000 CNY (33.1%) and >6,000 CNY (15.2%, *p* < 0.001). Urban residents were significantly more likely to have documented screening than rural residents (72.4% vs. 47.1%, *p* < 0.001). Similarly, medical insurance coverage was a strong positive factor, with insured women far more likely to be screened than uninsured women (93.8% vs. 64.4%, p < 0.001). Behavioral and healthcare access variables obtained from routine health examination records also showed significant differences by screening status. Non-smokers had higher screening rates (84.8%) compared with current (5.5%) and former smokers (9.7%, *p* = 0.015). Physical activity level was positively associated with screening, with women engaging in ≥3 sessions of moderate-to-vigorous activity per week showing the highest participation (30.3%) compared with those engaging 1–2 times (47.6%) or none (22.1%, *p* = 0.002). Accessibility of healthcare services played an additional role: women residing ≤5 km from a medical facility had the highest screening participation (64.1%), whereas those living >15 km away were least likely to be screened (6.9%, *p* < 0.001). A documented history of sexually transmitted infections (STIs) was slightly less frequent among screened women (6.2%) than among unscreened women (8.0%, *p* = 0.038).

**Table 1:** Baseline characteristics of the study population (*N* = 2,320).

Characteristic	Total (*n* = 2,320)	Screened (*n* = 1,450)	Not screened (*n* = 870)	*p*-value
Age, mean (SD)	38.5 (8.6)	37.8 (8.3)	39.8 (8.9)	<0.001
Age group, *n* (%)				<0.001
< 30 years	420 (18.1)	300 (20.7)	120 (13.8)	
30–39 years	920 (39.7)	610 (42.1)	310 (35.6)	
40–49 years	720 (31.0)	410 (28.3)	310 (35.6)	
≥ 50 years	260 (11.2)	130 (9.0)	130 (14.9)	
Ethnicity, *n* (%)				<0.001
Han	1860 (80.2)	1,220 (84.1)	640 (73.6)	
Ethnic minority	460 (19.8)	230 (15.9)	230 (26.4)	
Education level, *n* (%)				<0.001
≤ Primary school	680 (29.3)	320 (22.1)	360 (41.4)	
Secondary school	1,020 (44.0)	680 (46.9)	340 (39.1)	
College or above	620 (26.7)	450 (31.0)	170 (19.5)	
Monthly income (CNY), *n* (%)				<0.001
< 3,000	920 (39.7)	480 (33.1)	440 (50.6)	
3000–6,000	1,060 (45.7)	750 (51.7)	310 (35.6)	
> 6,000	340 (14.7)	220 (15.2)	120 (13.8)	
Residence, *n* (%)				<0.001
Urban	1,460 (62.9)	1,050 (72.4)	410 (47.1)	
Rural	860 (37.1)	400 (27.6)	460 (52.9)	
Has medical insurance, *n* (%)				<0.001
Yes	1920 (82.8)	1,360 (93.8)	560 (64.4)	
No	400 (17.2)	90 (6.2)	310 (35.6)	
Smoking status, *n* (%)				0.015
Non-smoker	1860 (80.2)	1,230 (84.8)	630 (72.4)	
Former smoker	240 (10.3)	140 (9.7)	100 (11.5)	
Current smoker	220 (9.5)	80 (5.5)	140 (16.1)	
Physical activity level, *n* (%)				0.002
None	620 (26.7)	320 (22.1)	300 (34.5)	
1–2 times per week	1,080 (46.6)	690 (47.6)	390 (44.8)	
≥ 3 times per week	620 (26.7)	440 (30.3)	180 (20.7)	
Distance to clinic, *n* (%)				<0.001
≤ 5 km	1,280 (55.2)	930 (64.1)	350 (40.2)	
6–15 km	740 (31.9)	420 (29.0)	320 (36.8)	
> 15 km	300 (12.9)	100 (6.9)	200 (23.0)	
History of STIs, *n* (%)				0.038
Yes	160 (6.9)	90 (6.2)	70 (8.0)	
No	2,160 (93.1)	1,360 (93.8)	800 (92.0)	

SD, standard deviation; CNY, Chinese Yuan; STIs, sexually transmitted infections. Values are presented as mean (SD) for continuous variables and *n* (%) for categorical variables. *p*-values are derived from *t*-tests for continuous variables and chi-squared tests for categorical variables. Statistically significant *p*-values (<0.05) indicate significant differences between the screened and not screened groups.

### Multivariate logistic regression analysis of factors associated with cervical cancer screening (*N* = 2,320)

Table 2 summarizes the results of the multivariate logistic regression model examining factors associated with cervical cancer screening participation. Increasing age was independently associated with lower screening participation, with each additional year of age decreasing the likelihood of screening by approximately 4% (adjusted odds ratio [aOR] = 0.96, 95% confidence interval [CI]: 0.94–0.98, *p* < 0.001). Ethnic minority women had substantially lower odds of documented screening compared with Han women (aOR = 0.61, 95% CI: 0.49–0.76, *p* < 0.001). Education level and household income, as recorded in the health information system, were both positively associated with screening participation. Compared with women who had primary education or below, those with secondary education had 52% higher odds of screening (aOR = 1.52, 95% CI: 1.26–1.83, *p* < 0.001), while women with a college education or above had more than twice the odds (aOR = 2.30, 95% CI: 1.84–2.87, p < 0.001). Similarly, women earning 3,000–6,000 CNY per month were 74% more likely to have a recorded screening compared with those earning <3,000 CNY (aOR = 1.74, 95% CI: 1.44–2.11, *p* < 0.001), and those earning > 6,000 CNY also showed significantly higher odds (aOR = 1.51, 95% CI: 1.14–2.00, *p* = 0.004). Urban residence showed a strong association with cervical cancer screening participation, with urban women being more than twice as likely to have undergone screening as rural women (aOR = 2.13, 95% CI: 1.78–2.54, *p* < 0.001). Medical insurance coverage represented the most influential factor: insured women had over fourfold higher odds of screening participation compared with uninsured women (aOR = 4.12, 95% CI: 3.19–5.33, *p* < 0.001). Health behavior variables, as documented during routine health assessments, were also associated with screening participation. Current smoking status was linked to a significantly lower likelihood of screening compared with non-smoking (aOR = 0.47, 95% CI: 0.33–0.67, p < 0.001), whereas former smoking showed no significant association (aOR = 0.92, 95% CI: 0.71–1.19, *p* = 0.520). Higher physical activity levels were positively correlated with screening participation, with women engaging in ≥3 sessions of moderate-to-vigorous exercise per week showing 48% greater odds of screening compared with inactive women (aOR = 1.48, 95% CI: 1.17–1.88, *p* = 0.001). Healthcare accessibility was inversely associated with screening likelihood. Women residing 6–15 km from the nearest clinic had 22% lower odds of screening (aOR = 0.78, 95% CI: 0.62–0.99, *p* = 0.042), and those living > 15 km away had 61% lower odds (aOR = 0.39, 95% CI: 0.28–0.54, *p* < 0.001), relative to women living within ≤ 5 km. A documented history of sexually transmitted infections (STIs) was not significantly associated with screening participation (aOR = 0.76, 95% CI: 0.53–1.10, *p* = 0.147). Overall, this retrospective analysis identified several independent predictors of cervical cancer screening participation. Sociodemographic characteristics (age, ethnicity, education, income, and residence), healthcare accessibility (insurance coverage and distance to a clinic), and health behaviors (smoking status and physical activity) showed significant associations with cervical cancer screening participation, suggesting that screening participation is shaped by multiple interrelated factors within this population.

**Table 2:** Multivariate logistic regression analysis of factors associated with cervical cancer screening (*N* = 2,320).

Variable	Adjusted odds ratio (aOR)	95% confidence interval (CI)	*p*-value
Age (per 1-year increase)	0.96	0.94–0.98	<0.001
Ethnicity			
Han (Ref)	1.00		
Ethnic minority	0.61	0.49–0.76	<0.001
Education level			
≤ Primary school (Ref)	1.00		
Secondary school	1.52	1.26–1.83	<0.001
College or above	2.30	1.84–2.87	<0.001
Monthly income (CNY)			
< 3,000 (Ref)	1.00		
3000–6,000	1.74	1.44–2.11	<0.001
> 6,000	1.51	1.14–2.00	0.004
Residence			
Urban	2.13	1.78–2.54	<0.001
Rural (Ref)	1.00		
Has medical insurance			
Yes	4.12	3.19–5.33	<0.001
No (Ref)	1.00		
Smoking status			
Non-smoker (Ref)	1.00		
Former smoker	0.92	0.71–1.19	0.520
Current smoker	0.47	0.33–0.67	<0.001
Physical activity level			
None (Ref)	1.00		
1–2 times per week	1.17	0.96–1.42	0.119
≥ 3 times per week	1.48	1.17–1.88	0.001
Distance to clinic			
≤ 5 km (Ref)	1.00		
6–15 km	0.78	0.62–0.99	0.042
> 15 km	0.39	0.28–0.54	<0.001
History of STIs			
No (Ref)	1.00		
Yes	0.76	0.53–1.10	0.147

Adjusted odds ratios (aORs) were obtained from a multivariate logistic regression model with cervical cancer screening status (screened vs. not screened) as the dependent variable. The model was adjusted for age, ethnicity, education level, monthly income, residence, medical insurance status, smoking status, physical activity level, distance to the nearest clinic, and history of sexually transmitted infections (STIs). Statistically significant *p*-values (<0.05) indicate a significant association between the variable and screening participation. Ref, Reference category; CNY, Chinese Yuan; STIs, sexually transmitted infections.

### Subgroup analysis of factors associated with cervical cancer screening by residence (urban vs. rural)

Table 3 presents the results of the subgroup logistic regression analyses examining factors associated with cervical cancer screening participation after stratification by residence (urban vs. rural). Interaction terms were tested to assess whether the strength of these associations differed between the two subgroups. Increasing age was inversely associated with screening participation in both urban and rural populations, with a slightly stronger negative association observed among urban residents (adjusted odds ratio [aOR] = 0.95, 95% confidence interval [CI]: 0.92–0.98) than among rural residents (aOR = 0.97, 95% CI: 0.94–1.00); however, the interaction test indicated no statistically significant difference between groups (*p* = 0.215). Ethnic minority status was consistently associated with lower odds of documented screening in both subgroups (urban: aOR = 0.68, 95% CI: 0.52–0.89; rural: aOR = 0.57, 95% CI: 0.41–0.78), and no significant interaction by residence was detected (*p* = 0.362). Education level, as recorded in the health information system, demonstrated a strong positive association with screening in both subgroups. Compared with women who had primary education or below, those with a college education or above had substantially higher odds of screening (urban: aOR = 2.15, 95% CI: 1.68–2.75; rural: aOR = 2.52, 95% CI: 1.75–3.63), with no significant interaction by residence (*p* = 0.307). Similarly, monthly income showed a positive association with screening participation in both urban and rural women, with the highest odds observed among those earning 3,000–6,000 CNY per month (urban: aOR = 1.68, 95% CI: 1.32–2.14; rural: aOR = 1.83, 95% CI: 1.29–2.61), and no significant interaction effect (*p* = 0.688). Medical insurance coverage remained a strong predictor of screening in both groups, as insured women had substantially higher odds of participation compared with uninsured women (urban: aOR = 3.86, 95% CI: 2.96–5.03; rural: aOR = 4.46, 95% CI: 2.91–6.85), and the interaction test confirmed no significant difference by residence (*p* = 0.476). Current smoking, based on routinely documented health behavior data, was inversely associated with screening participation in both subgroups (urban: aOR = 0.50, 95% CI: 0.29–0.87; rural: aOR = 0.44, 95% CI: 0.27–0.72), with no interaction effect (*p* = 0.781). Physical activity level, also derived from standardized health examination records, was positively associated with screening participation in both urban (aOR = 1.46, 95% CI: 1.08–1.96) and rural (aOR = 1.51, 95% CI: 1.06–2.16) populations, again without significant interaction by residence (*p* = 0.871). Distance to the nearest healthcare facility showed a consistent inverse association with screening across both subgroups. Women residing >15 km from a clinic had substantially lower odds of screening compared with those living within ≤5 km (urban: aOR = 0.37, 95% CI: 0.25–0.53; rural: aOR = 0.40, 95% CI: 0.26–0.61), and the difference between subgroups was not statistically significant (*p* = 0.812).

**Table 3:** Subgroup analysis of factors associated with cervical cancer screening by residence (urban vs. rural).

Variable	Urban (*n* = 1,460)	Rural (*n* = 860)	*p*-value for interaction
Age (per 1-year increase)	0.95 (0.92–0.98)	0.97 (0.94–1.00)	0.215
Ethnicity			
Han (Ref)	1.00	1.00	
Ethnic minority	0.68 (0.52–0.89)	0.57 (0.41–0.78)	0.362
Education level			
≤ Primary school (Ref)	1.00	1.00	
Secondary school	1.40 (1.12–1.75)	1.61 (1.19–2.18)	0.523
College or above	2.15 (1.68–2.75)	2.52 (1.75–3.63)	0.307
Monthly income (CNY)			
< 3,000 (Ref)	1.00	1.00	
3000–6,000	1.68 (1.32–2.14)	1.83 (1.29–2.61)	0.688
> 6,000	1.49 (1.05–2.11)	1.54 (0.94–2.51)	0.902
Has medical insurance			
Yes	3.86 (2.96–5.03)	4.46 (2.91–6.85)	0.476
No (Ref)	1.00	1.00	
Smoking status			
Non-smoker (Ref)	1.00	1.00	
Former smoker	0.88 (0.61–1.26)	1.02 (0.72–1.45)	0.642
Current smoker	0.50 (0.29–0.87)	0.44 (0.27–0.72)	0.781
Physical activity level			
None (Ref)	1.00	1.00	
1–2 times per week	1.12 (0.89–1.41)	1.24 (0.91–1.69)	0.553
≥ 3 times per week	1.46 (1.08–1.96)	1.51 (1.06–2.16)	0.871
Distance to clinic			
≤ 5 km (Ref)	1.00	1.00	
6–15 km	0.80 (0.63–1.02)	0.76 (0.52–1.11)	0.744
> 15 km	0.37 (0.25–0.53)	0.40 (0.26–0.61)	0.812

Adjusted odds ratios (aORs) and 95% confidence intervals (CIs) were calculated using multivariate logistic regression models stratified by residence (urban vs. rural). The dependent variable is cervical cancer screening status (screened vs. not screened). All models were adjusted for age, ethnicity, education level, monthly income, medical insurance status, smoking status, physical activity level, and distance to the nearest clinic. The *p*-value for interaction tests whether the effect of each variable on screening participation differs significantly between urban and rural residents. Ref, Reference category; CNY, Chinese Yuan.

### Sensitivity analysis of factors associated with cervical cancer screening under different model assumptions

Table 4 summarizes the results of sensitivity analyses conducted to evaluate the robustness of factors associated with cervical cancer screening participation under three analytic scenarios: complete-case analysis (Model 1), adjustment for additional covariates such as marital status and parity (Model 2), and multiple imputation for missing data (Model 3). The findings were highly consistent across all models, confirming the stability of the associations identified in the main analysis. Age remained negatively associated with screening participation in all models, with each additional year of age reducing the odds of screening by approximately 4% (Model 1: aOR = 0.96, 95% CI 0.94–0.98; Model 2: aOR = 0.95, 95% CI 0.93–0.98; Model 3: aOR = 0.96, 95% CI 0.94–0.98; p for robustness = 0.721). Ethnicity showed a similarly stable association, as ethnic minority women consistently exhibited lower odds of documented screening compared with Han women (Model 1: aOR = 0.61, 95% CI 0.49–0.76; Model 2: aOR = 0.62, 95% CI 0.50–0.77; Model 3: aOR = 0.60, 95% CI 0.48–0.75; p for robustness = 0.893). Education level retained a strong positive association with screening participation, with higher education corresponding to increased screening likelihood. Compared with women who had primary education or below, those with secondary education and college education or above remained significantly more likely to undergo screening, with minimal variation across models (e.g., college or above: Model 1: aOR = 2.30, 95% CI 1.84–2.87; Model 2: aOR = 2.28, 95% CI 1.82–2.85; Model 3: aOR = 2.31, 95% CI 1.85–2.89; p for robustness = 0.912). Similarly, monthly income demonstrated a consistent positive gradient, with women earning 3,000–6,000 CNY per month showing the highest screening participation across all models (Model 1: aOR = 1.74, 95% CI 1.44–2.11; Model 2: aOR = 1.73, 95% CI 1.43–2.10; Model 3: aOR = 1.75, 95% CI 1.45–2.12; p for robustness = 0.947). Medical insurance coverage remained the strongest predictor of screening participation in each model, with insured women having more than fourfold higher odds of documented screening compared with uninsured women (Model 1: aOR = 4.12, 95% CI 3.19–5.33; Model 2: aOR = 4.08, 95% CI 3.16–5.27; Model 3: aOR = 4.15, 95% CI 3.21–5.36; p for robustness = 0.984). Health behavior variables derived from routine health examination data also exhibited consistent associations. Current smoking was associated with a substantially lower likelihood of screening across all models (Model 1: aOR = 0.48, 95% CI 0.33–0.69; Model 2: aOR = 0.46, 95% CI 0.32–0.67; Model 3: aOR = 0.47, 95% CI 0.32–0.68; p for robustness = 0.893), whereas former smoking was not significant. Physical activity showed a positive and stable relationship with screening participation, particularly among women engaging in ≥3 sessions of moderate-to-vigorous activity per week (Model 1: aOR = 1.46, 95% CI 1.16–1.84; Model 2: aOR = 1.45, 95% CI 1.15–1.82; Model 3: aOR = 1.47, 95% CI 1.17–1.85; p for robustness = 0.934).

**Table 4:** Sensitivity analysis of factors associated with cervical cancer screening under different model assumptions.

Variable	Model 1: complete case analysis	Model 2: adjusted for additional covariates	Model 3: multiple imputation analysis	*p*-value for robustness
Age (per 1-year increase)	0.96 (0.94–0.98)	0.95 (0.93–0.98)	0.96 (0.94–0.98)	0.721
Ethnicity				
Han (Ref)	1.00	1.00	1.00	
Ethnic Minority	0.61 (0.49–0.76)	0.62 (0.50–0.77)	0.60 (0.48–0.75)	0.893
Education level				
≤ Primary school (Ref)	1.00	1.00	1.00	
Secondary school	1.52 (1.26–1.83)	1.50 (1.24–1.82)	1.53 (1.27–1.84)	0.984
College or above	2.30 (1.84–2.87)	2.28 (1.82–2.85)	2.31 (1.85–2.89)	0.912
Monthly income (CNY)				
< 3,000 (Ref)	1.00	1.00	1.00	
3000–6,000	1.74 (1.44–2.11)	1.73 (1.43–2.10)	1.75 (1.45–2.12)	0.947
> 6,000	1.51 (1.14–2.00)	1.49 (1.12–1.98)	1.52 (1.15–2.02)	0.911
Has medical insurance				
Yes	4.12 (3.19–5.33)	4.08 (3.16–5.27)	4.15 (3.21–5.36)	0.984
No (Ref)	1.00	1.00	1.00	
Smoking status				
Non-smoker (Ref)	1.00	1.00	1.00	
Former smoker	0.91 (0.69–1.20)	0.90 (0.68–1.19)	0.92 (0.70–1.21)	0.814
Current smoker	0.48 (0.33–0.69)	0.46 (0.32–0.67)	0.47 (0.32–0.68)	0.893
Physical activity level				
None (Ref)	1.00	1.00	1.00	
1–2 times per week	1.15 (0.93–1.42)	1.14 (0.92–1.42)	1.16 (0.93–1.43)	0.978
≥ 3 times per week	1.46 (1.16–1.84)	1.45 (1.15–1.82)	1.47 (1.17–1.85)	0.934

Model 1 represents a complete case analysis excluding individuals with missing data; Model 2 adjusts for additional covariates, including marital status and parity, to test the robustness of associations; Model 3 uses multiple imputation to handle missing data, with 20 imputations performed. Adjusted odds ratios (aORs) and 95% confidence intervals (CIs) were derived from logistic regression models. All models were adjusted for age, ethnicity, education level, monthly income, residence, medical insurance status, smoking status, and physical activity level. The *p*-value for robustness tests whether the associations remain consistent across the three models. Ref, Reference category; CNY, Chinese Yuan.

## Discussion

This retrospective cross-sectional analysis explored associations between sociodemographic, behavioral, and healthcare-related factors and cervical cancer screening participation among women in Southwest China. Based on systematically retrieved medical and screening records, the study found that disparities in screening participation were evident within this sample of women with documented healthcare encounters, and these disparities were associated with age, ethnicity, education, income, residence, health behaviors, and healthcare accessibility. These findings underscore the existence of substantial inequities within routine screening programs and highlight the importance of strengthening targeted, population-specific strategies to improve coverage—particularly among socioeconomically disadvantaged and geographically remote groups.

Age was a significant predictor of screening participation, with younger women being more likely to undergo cervical cancer screening than older women. This aligns with previous research indicating that older women often perceive themselves at lower risk, may have misconceptions about screening necessity, or encounter physical or logistical barriers to accessing services (15, 16). Given that the incidence of cervical cancer increases with age, efforts to promote screening uptake among older women should focus on risk communication and age-specific outreach strategies.

Ethnicity also played a critical role, with ethnic minority women exhibiting significantly lower screening participation rates compared to Han women. This finding is consistent with prior studies showing that cultural and linguistic barriers may deter ethnic minorities from engaging in preventive healthcare services (17, 18). Language differences, health literacy challenges, and sociocultural norms likely contribute to disparities in screening uptake (19). Interventions should consider culturally tailored health education programs and increased availability of bilingual healthcare providers to bridge this gap.

Women with lower education and income were less likely to participate in cervical cancer screening; however, the underlying reasons for this association could not be directly assessed in this study (20). Policies aimed at providing financial assistance, increasing awareness campaigns, and integrating screening services into routine primary care could help mitigate these disparities.

Our study found that urban residence and proximity to healthcare facilities were significantly associated with cervical cancer screening participation. Women living in urban areas were more than twice as likely to undergo screening compared to their rural counterparts. Geographic remoteness and inadequate healthcare infrastructure in rural regions may contribute to lower screening rates, consistent with prior Chinese evidence derived from different study designs, including population-based analyses using nationally representative survey data [e.g., Liu et al. (21)] as well as hospital- or platform-based cross-sectional studies relying on convenience samples [e.g., Zhang et al. (22)]; these differences in sampling frameworks should be considered when comparing effect estimates and generalizability. Women living more than 15 km from a healthcare facility had the lowest screening rates, highlighting the importance of improving healthcare accessibility in remote areas. Expanding mobile screening units, telemedicine consultations, and transportation assistance programs could be effective strategies to address these barriers.

Medical insurance coverage showed the strongest association with cervical cancer screening participation, with insured women being over four times more likely to be screened than uninsured women. This finding is consistent with previous studies reporting an association between financial coverage and greater utilization of preventive care services (23). Despite the availability of national screening programs, gaps in healthcare coverage persist, particularly among marginalized groups. Policymakers should consider expanding insurance coverage for preventive services and reducing out-of-pocket costs to increase screening uptake.

Health behaviors such as smoking and physical activity also played a role in screening participation. Current smokers were significantly less likely to undergo screening, a finding consistent with previous studies suggesting that smokers may be less engaged in preventive healthcare, which may partly reflect differences in engagement with preventive healthcare (12, 24). Given that smoking is a known risk factor for cervical cancer, targeted health education campaigns emphasizing the dual benefits of smoking cessation and cervical cancer prevention may be beneficial.

Conversely, regular physical activity was associated with increased screening participation, indicating that women who engage in healthier lifestyles may also be more proactive about preventive healthcare. This trend suggests that health promotion programs encouraging an active lifestyle could integrate messages about cervical cancer screening to reinforce positive health behaviors (25).

This study has several strengths, including its large sample size, rigorous multistage sampling method, and robust statistical analyses. These findings offer insights into sociodemographic, behavioral, and healthcare-related factors associated with cervical cancer screening participation in Southwest China. The inclusion of subgroup and sensitivity analyses further strengthens the reliability and generalizability of our results.

Several limitations of this retrospective analysis should be considered when interpreting the findings. First, although all data were obtained from institutional medical records and regional screening databases, some variables—particularly lifestyle- and behavior-related indicators—were derived from archived health assessment forms completed during routine visits. These data were not originally collected for research purposes and may therefore be subject to reporting inaccuracies or incomplete entries. Despite data validation and logical cross-checking procedures, residual misclassification bias cannot be entirely excluded. In addition, screening participation was ascertained from administrative and laboratory records, which reflect healthcare utilization rather than individual intention or awareness; consequently, some women who underwent screening outside the local healthcare system may have been misclassified as unscreened. Second, the study population was derived from routine outpatient and community health encounter databases; therefore, women with no documented contact with the healthcare system during the study period were not captured. This selection mechanism may limit the generalizability of the findings to the broader community and could lead to an underestimation of socioeconomic and geographic barriers to cervical cancer screening, as the “not screened” group in this analysis represents non-participation among healthcare users rather than women completely disconnected from care. Importantly, the observed disparities were evident even within this population of healthcare users. Third, the retrospective cross-sectional design inherently limits causal inference. Because exposure and outcome data were derived from the same time window, temporal relationships between sociodemographic, behavioral, and healthcare access factors and screening participation cannot be definitively established. Accordingly, the observed associations should be interpreted as correlational rather than causal. Future prospective or longitudinal studies are warranted to clarify temporal relationships and to explore potential mediating pathways, such as health literacy, social support, and health system navigation. Fourth, although the sample size was relatively large and drawn from Southwest China, the study population may not fully capture regional heterogeneity across other parts of the country. Variations in healthcare delivery models, insurance policies, resource allocation, and cultural norms may affect the external generalizability of the findings. In addition, some potentially relevant factors—such as partner support, reproductive history, and awareness of HPV vaccination—were not available in the databases and could not be evaluated. Finally, although rigorous statistical methods, including multivariable adjustment, subgroup analyses, and sensitivity analyses, were applied to enhance internal validity, the use of routinely collected administrative data is inherently subject to limitations such as coding errors, missing variables, and incomplete documentation. Future research integrating multi-center data sources, prospective follow-up, and qualitative assessments of healthcare-seeking behavior may provide a more comprehensive understanding of cervical cancer screening disparities in diverse Chinese populations.

This retrospective study highlights the need for integrated strategies to enhance cervical cancer screening coverage and reduce disparities in Southwest China. Policy efforts should prioritize health education and culturally tailored communication to improve awareness and participation among older women and ethnic minorities. Expanding healthcare accessibility through mobile screening services and strengthened primary care infrastructure is essential to reach rural and remote populations. Financial interventions, including broader insurance reimbursement and subsidized screening for low-income women, could further mitigate economic barriers. Integrating screening promotion into existing health campaigns—such as physical activity and smoking cessation programs—may also reinforce preventive health behaviors. At the regional level, effective implementation requires coordination within the established Maternal and Child Health (MCH) network. Leveraging community health workers and family physicians as key outreach agents can improve local engagement and follow-up. Strengthening the “Two Cancers Screening Program” through targeted funding, intersectoral collaboration, and culturally adaptive delivery models offers a feasible and sustainable path forward. Although the retrospective cross-sectional design limits causal inference, these findings provide evidence-based guidance for policy and practice aimed at promoting equitable screening participation. Future longitudinal and interventional studies are warranted to confirm these associations and to evaluate the long-term effectiveness of targeted screening strategies across diverse populations in China.

This study found that disparities in cervical cancer screening participation were evident even within this sample of women with documented healthcare encounters. Despite the existence of nationwide screening programs, gaps in service reach, financial protection, and public awareness remain evident—particularly among rural residents, ethnic minorities, and women with limited income or education. Strengthening targeted outreach, expanding insurance reimbursement, and improving accessibility to primary screening services are critical steps toward equitable coverage and effective cervical cancer prevention in Southwest China. Further longitudinal and interventional studies are warranted to confirm these retrospective associations and to elucidate the underlying causal mechanisms shaping screening behavior.

## Data Availability

The original contributions presented in the study are included in the article/supplementary material, further inquiries can be directed to the corresponding authors.

## References

[1] Singh D Vignat J Lorenzoni V Eslahi M Ginsburg O Lauby-Secretan B . Global estimates of incidence and mortality of cervical cancer in 2020: a baseline analysis of the WHO global cervical Cancer elimination initiative. Lancet Glob Health. (2023) 11:e197–206. doi: 10.1016/S2214-109X(22)00501-0, 36528031 PMC9848409

[2] Okunade KS. Human papillomavirus and cervical cancer. J Obstet Gynaecol. (2020) 40:602–8. doi: 10.1080/01443615.2019.1634030, 31500479 PMC7062568

[3] Shapiro GK. HPV vaccination: an underused strategy for the prevention of Cancer. Curr Oncol. (2022) 29:3780–92. doi: 10.3390/curroncol29050303, 35621693 PMC9140027

[4] Wirtz C Mohamed Y Engel D Sidibe A Holloway M Bloem P . Integrating HPV vaccination programs with enhanced cervical cancer screening and treatment, a systematic review. Vaccine. (2022) 40:A116–23. doi: 10.1016/j.vaccine.2021.11.013, 34863615

[5] Zhao Y Bao H Ma L Song B di J Wang L . Real-world effectiveness of primary screening with high-risk human papillomavirus testing in the cervical cancer screening programme in China: a nationwide, population-based study. BMC Med. (2021) 19:164. doi: 10.1186/s12916-021-02026-0, 34261463 PMC8281674

[6] Ma Y Di J Bi H Zhao Q Qin T Xu W . Comparison of the detection rate of cervical lesion with truscreen, LBC test and HPV test: a real-world study based on population screening of cervical cancer in rural areas of China. PLoS One. (2020) 15:e0233986. doi: 10.1371/journal.pone.0233986, 32634143 PMC7340311

[7] Gong X Xu J He Y Zou G Liu J. Socioeconomic inequalities in human papillomavirus knowledge and vaccine uptake: evidence from a cross-sectional study in China. Front Public Health. (2024) 12:1399192. doi: 10.3389/fpubh.2024.1399192, 38993697 PMC11236539

[8] Zhang W Gao K Fowkes FJI Adeloye D Rudan I Song P . Associated factors and global adherence of cervical cancer screening in 2019: a systematic analysis and modelling study. Glob Health. (2022) 18:101. doi: 10.1186/s12992-022-00890-w, 36494856 PMC9733311

[9] Alshammari AH Ishii H Hirotsu T Hatakeyama H Morishita M di Luccio E. Bridging the gap in cervical cancer screening for underserved communities: MCED and the promise of future technologies. Front Oncol. (2024) 14:1407008. doi: 10.3389/fonc.2024.1407008, 39135996 PMC11317246

[10] Petersen Z Jaca A Ginindza TG Maseko G Takatshana S Ndlovu P . Barriers to uptake of cervical cancer screening services in low-and-middle-income countries: a systematic review. BMC Womens Health. (2022) 22:2. doi: 10.1186/s12905-022-02043-y, 36461001 PMC9716693

[11] Suk R Hong YR Rajan SS Xie Z Zhu Y Spencer JC. Assessment of US preventive services task force guideline-concordant cervical Cancer screening rates and reasons for Underscreening by age, race and ethnicity, sexual orientation, rurality, and insurance, 2005 to 2019. JAMA Netw Open. (2022) 5:e2143582. doi: 10.1001/jamanetworkopen.2021.43582, 35040970 PMC8767443

[12] Chang HK Myong JP Byun SW Lee SJ Lee YS Lee HN . Factors associated with participation in cervical cancer screening among young Koreans: a nationwide cross-sectional study. BMJ Open. (2017) 7:e013868. doi: 10.1136/bmjopen-2016-013868, 28373252 PMC5387966

[13] Yan H Wang Q Qiao Y. Cervical cancer prevention in China: where are we now, and what's next? Cancer Biol Med. (2024) 21:213–7. doi: 10.20892/j.issn.2095-3941.2023.0432, 38172555 PMC10976326

[14] Farajimakin O. Barriers to cervical Cancer screening: a systematic review. Cureus. (2024) 16:e65555. doi: 10.7759/cureus.65555, 39192892 PMC11347962

[15] Marlow LAV Ryan M Waller J. Increasing the perceived relevance of cervical screening in older women who do not plan to attend screening. Sex Transm Infect. (2020) 96:20–5. doi: 10.1136/sextrans-2019-054120, 31395750 PMC7029243

[16] Miller TA Flowers L. Navigating the cervical cancer screening guidelines for women aged older than 65 years. Menopause. (2017) 24:1302–3. doi: 10.1097/GME.0000000000000913, 28697040

[17] Zhao W So WKW Li H Wong CL. Knowledge, attitudes, and practices regarding cancer screening and primary prevention among ethnic minorities in mainland China: a literature review. Asia Pac J Oncol Nurs. (2024) 11:100435. doi: 10.1016/j.apjon.2024.100435, 38601109 PMC11004070

[18] Lin W Chen B Wu B Yuan S Zhong C Huang W . Cervical Cancer screening rate and willingness among female migrants in Shenzhen, China: three-year changes in citywide surveys. Cancer Res Treat. (2021) 53:212–22. doi: 10.4143/crt.2020.219, 32878425 PMC7812020

[19] Afsah YR Kaneko N. Barriers to cervical cancer screening faced by immigrant Muslim women: a systematic scoping review. BMC Public Health. (2023) 23:2375. doi: 10.1186/s12889-023-17309-9, 38037019 PMC10687813

[20] Murfin J Irvine F Meechan-Rogers R Swift A. Education, income and occupation and their influence on the uptake of cervical cancer prevention strategies: a systematic review. J Clin Nurs. (2020) 29:393–415. doi: 10.1111/jocn.15094, 31713934

[21] Liu Y Guo J Zhu G Zhang B Feng XL. Changes in rate and socioeconomic inequality of cervical cancer screening in northeastern China from 2013 to 2018. Front Med (Lausanne). (2022) 9:913361. doi: 10.3389/fmed.2022.913361, 36275788 PMC9580066

[22] Zhang B Wang S Yang X Chen M Ren W Bao Y . Knowledge, willingness, uptake and barriers of cervical cancer screening services among Chinese adult females: a national cross-sectional survey based on a large e-commerce platform. BMC Womens Health. (2023) 23:435. doi: 10.1186/s12905-023-02554-2, 37592252 PMC10436426

[23] Ozkardes C Harman J. Association of a patient's type of insurance with preventive service delivery. Cureus. (2023) 15:e44927. doi: 10.7759/cureus.44927, 37818517 PMC10560607

[24] Mansour MB Crone MR Sert E van Weert HC Chavannes NH van Asselt KM. Smoking cessation strategy in the national cervical cancer screening program (SUCCESS): study protocol for a pragmatic cluster randomised trial and process evaluation in Dutch general practice. BMJ Open. (2022) 12:e055812. doi: 10.1136/bmjopen-2021-055812, 35379626 PMC8981275

[25] Villalobos A Chambers DA. Advancing the science of integrating multiple interventions by blending and bundling. JNCI Cancer Spectr. (2023) 7:pkad070. doi: 10.1093/jncics/pkad070, 37707597 PMC10587993

